# Injectable Devices for Delivery of Liquid or Solid Protein Formulations

**DOI:** 10.1021/acsmaterialsau.3c00004

**Published:** 2023-03-13

**Authors:** Daniel A. Bernards, Chu Jian Ma, Youning Zhang, Tannia M. Rodriguez, John Dickson, Bhushan N. Kharbikar, Robert B. Bhisitkul, Tejal A. Desai

**Affiliations:** † University of California, San Francisco, Department of Bioengineering and Therapeutic Sciences, San Francisco, California 94143, United States; ‡ University of California, San Francisco, Department of Ophthalmology, San Francisco, California 94143, United States; § Brown University, School of Engineering, Providence, Rhode Island 02912, United States

**Keywords:** drug delivery, protein, biologic, polycaprolactone, injectable, ocular

## Abstract

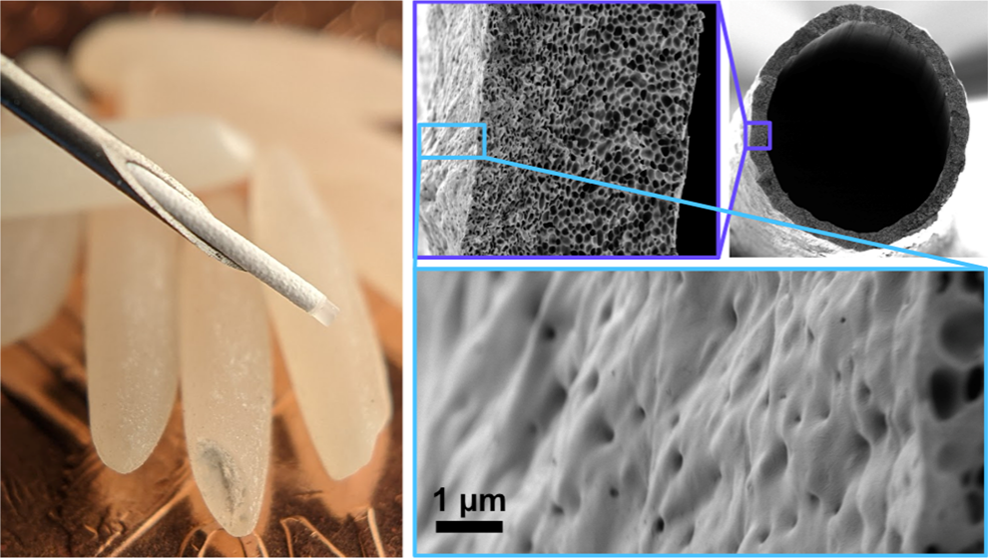

Sustained delivery of protein therapeutics remains a largely unsolved problem across anatomic locations. Miniaturized devices that can provide sustained delivery of protein formulations have the potential to address this challenge via minimally invasive administration. In particular, methodologies that can optimize protein formulation independent of device manufacture have the greatest potential to provide a platform suitable for wide applications. The techniques developed here demonstrate the fabrication of tubular devices for sustained release of protein therapeutics. Utilizing a dip-casting process, fine-scale tubes can be reliably produced with wall thickness down to 30 μm. Techniques were developed that enabled effective loading of either solid or liquid formulations, while maintaining a cylindrical form-factor compatible with placement in a 22-gauge needle. Further, highly compacted protein pellets that approach the expected density of the raw materials were produced with a diameter (∼300 μm) suitable for miniaturized devices. Release from a solid-loaded device was capable of sustaining release of a model protein in excess of 400 days. Given significant interest in ocular applications, intravitreal injection was demonstrated in a rabbit model with these devices. In addition, to simulate repeated injections in ocular applications, serial intravitreal injection of two devices in a rabbit model demonstrated acceptable ocular safety without significant intraocular inflammation from clinical exam and histology.

## Introduction

Long-term drug delivery has historically been dominated by devices releasing small molecule therapeutics.^1^ Particle-based or monolithic devices delivering small molecules benefit in a number of ways, such as well-characterized release models (e.g., Higuchi model)^2^ or compatibility with industrial manufacturing processes (e.g., emulsion or extrusion).^3,4^ However, a majority of active pharmaceutical ingredients (APIs) in such release platforms nonspecifically target complex anti-inflammatory, pain, or hormonal pathways.^5^ Biologic therapeutics conversely target highly specific biomolecular pathways of disease, which are known to minimize or fully avoid off-target interactions. This comes with a trade off as biologics are complex macromolecules that have limited transport across biological barriers and typically need to be administered systemically (intravenously or subcutaneously) or directly at the site of action (e.g., intraocularly).^6^ Furthermore, because biologics are typically administered as a bolus dose, large excesses are typically used and a majority of the administered dose is cleared with diminished efficacy.

Inefficiencies in delivery make biologics an attractive target for drug delivery technologies, yet the unique nature of biologics limits the direct application of technologies used for small-molecule delivery. Compared to small molecules that can be synthetically produced and structurally verified, biologics typically require specific secondary and tertiary structures to be efficacious. Consequently, biologic stability is generally more nuanced than that of small molecules and is more prone to incompatibility with conventional physical and chemical processing used in the fabrication of drug delivery devices. For any given biologic, physical and chemical stability, tendency for aggregation, or freeze–thaw behavior must all be investigated and may require formulation optimization to mitigate any shortcomings.^7^ Processes of denaturing, fragmentation, and aggregation can be impacted by protein concentrations, solution pH or osmolarity, solvent–protein interactions, or even physical pressure.^8,9^ Whether administered by injection,^10^ with a metered pump,^11^ or a permanent implant,^12^ delivery of biologics commonly relies on aqueous formulations of drug, where preparation of the administered drug is separated from its mode of delivery.

Intravenous or subcutaneous administration is well suited for systemic delivery of liquid formulations of biologics as it accommodates the large mass of drug required for systemic therapy. Yet many indications are localized and could benefit from more directed administration. In particular, delicate anatomical structures or confined areas for delivery, such as joints, the nasal cavity, or the eye, make compelling and challenging areas of development. Intra-articular cell-based biologic therapies for knee osteoarthritis have seen positive results that are albeit short-lived,^13^ which suggests such therapies can benefit from improved therapeutic residence time locally. For chronic sinusitus with nasal polyps, the biologic dupilumab has clinical efficacy when dosed subcutaneously, where systemic exposure and side effects would plausibly benefit from localized delivery.^14^ Lastly, treatment for retinal disease continues to rely on local delivery of biologics to the vitreous chamber and remains an open challenge in ophthalmology.

Ophthalmology has long been a target for drug delivery technology development given the rapid surface clearance and physical barriers to drug delivery.^15,16^ With the advent of anti-VEGF therapeutics requiring regular injections for the treatment of retinal disease, the field has pursued delivery solutions to reduce clinician burden while improving patient comfort and compliance. The Port Delivery System (PDS), developed specifically for sustained delivery of ranibizumab, achieved FDA approval in 2021 and represents the first sustained-delivery device for an ocular protein therapeutic.^12^ Placed in the vitreous and anchored to the sclera by a surgical procedure, the PDS enables patients to receive a single dose of concentrated ranibizumab (100 mg/mL) with a prescribed refill every 24 weeks, and an external port allows access for refilling upon depletion.^17^ Entry into the market is early and the recent voluntary recall bring about a murky outlook,^18^ but the underlying desire to achieve long-term biologic delivery with less frequent interventions is clear.

While effective, the above devices have limitations in terms of bulkiness or the drawbacks of a surgical procedure. Efforts to develop miniaturized and minimally invasive devices have been a goal of many drug delivery research efforts. In particular, degradable polymers, such as poly(lactic-*co*-glycolic acid) (PLGA) or poly(caprolactone) (PCL), are attractive since they can potentially obviate removal at the end of a device’s useful lifetime.^19−21^ PLGA is by far the most established degradable material and benefits from a record of application across physiological sites of action. Based on copolymer composition, its degradation time course can be modulated based on a target product profile. Further, PLGA comprises the matrix of Ozurdex, the first approved miniaturized device capable of injection by a fine-gauge needle, which provides steroid therapy to the eye with a specialized 22-gauge injector.^22^ However, PLGA is not without concern given the known inflammatory response to its degradation byproducts. For example, PLGA microspheres have been seen to exhibit significant toxicity in the eye.^23^ PCL is an alternative that degrades over months to years and offers a unique degradation profile, where PCL maintains structural integrity throughout the majority of the molecular degradation processes.^24^ This behavior can be advantageous for long-term drug delivery if release is dictated by material structure.

Prior work of Desai and colleagues has focused on utilizing thin porous PCL films that act as a rate-controlling membranes in sustained release devices.^25−28^ Lance et al. demonstrated sustained release of ranibizumab from a planar device architecture that released for 16 weeks.^25^ Follow-on work by Schlesinger et al. investigated release of aflibercept and transitioned to a roughly cylindrical form factor with release through 10 weeks.^26^ This work also demonstrated the ability to further formulate the payload of a reservoir-based device to improve the long-term stability of the loaded protein. While this development trended toward a device optimized for needle injection, the flat seals along the device and at each end prevented seamless insertion and deployment by needle. The ca. 1 mm diameter also required significant miniaturization to reach the widely accepted upper limit established by Ozurdex, which employs a 22-gauge thin-walled injector with an inner diameter of approximately 500 μm.^29^

The work of Jiang et al. surpassed these efforts by demonstrating a tubular PCL device architecture for delivery of protein therapeutics that could fit within the bore of a 21-gauge needle (nominally equivalent to a thin-walled 22-gauge needle).^30^ Devices were based on an electrospun tubular construct of chitosan and polycaprolactone, and injection of devices was demonstrated in an ex vivo porcine eye model. Tubes with a wall thickness of approximately 90 μm were demonstrated, and devices were filled by syringe with liquid or slurry formulations. Given the small device size, the wall thickness was significant and contributes to approximately 65% of the cross-sectional area of the device; further, being constrained to liquid-based formulations may limit broader translation and present challenges with storage. More recently Waterkotte et al. demonstrated a methodology to fabricate tubular devices from a flat film of PCL, where devices were heat and superglue sealed to fabricate liquid-loaded devices.^31^ Devices released in excess of 1 mg of bevacizumab over 90 days, but the greater payload correlated with a larger 946 μm diameter device, which is expected to require an 18-gauge or larger needle for delivery by injection. In addition, the superglue used to prevent leakage should be carefully assessed given potential cytotoxicity concerns associated with cyanoacrylates.^32^

In this work, we demonstrate the fabrication of tubular devices for the delivery of protein therapeutics, where the emphasis was implementing methods to achieve uniformly cylindrical devices suitable for deployment by injection. Devices based on dip-cast tubes enable a reliable wall thickness down to 30 μm, which improves the available reservoir volume for drug loading. Devices may be loaded with either a solid or liquid formulation, and a method to generate highly compacted pellets of protein with a diameter of 300 μm provide a means to maximize the loading capacity of such devices. Devices can be capped either by self-sealing the tubular construct or using a plug of solid polymer. These techniques to cap devices can generate a fully cylindrical form factor and avoid a distortion at either device end that may inhibit insertion and deployment by needle. Injection via a 22G needle is demonstrated both in vitro and in vivo. Safe, repeated intraocular injection was demonstrated with placebo devices, establishing a positive safety profile for multiple such devices present within the eye.

## Fabrication of Miniaturized PCL Devices

### Dip-Casting Thin-Walled PCL Tubes

**Figure 1:**
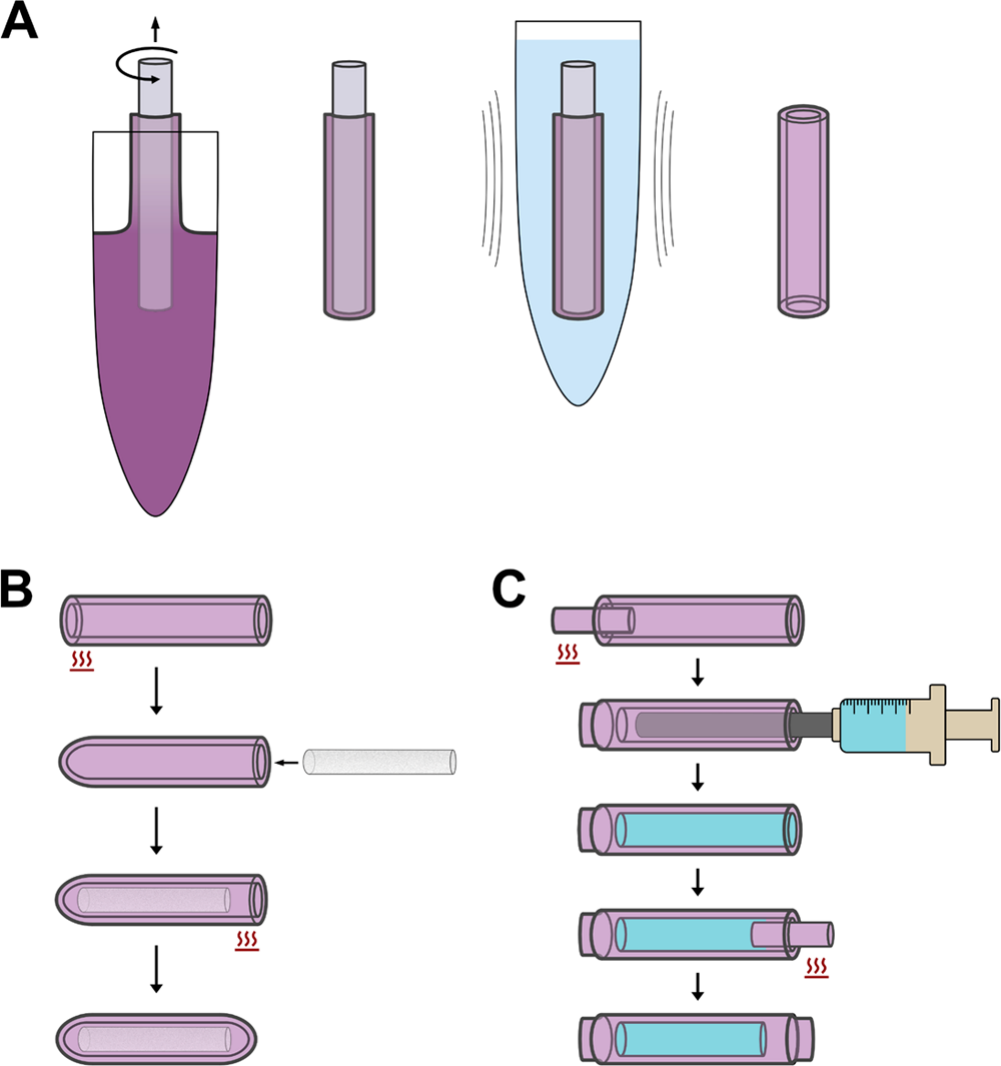
Fabrication of miniaturized tubular devices. (A) Tubes for device fabrication were dip-cast from PCL-porogen solution, air-dried of solvent, sonicated in release media, and mechanically removed. (B) Solid-loaded devices were fabricated by capping one tube end by self-sealing, inserting a precompacted pellet of payload, and self-sealing the remaining end. (C) Liquid-loaded devices were fabricated by capping one tube end with a solid filament, loading liquid into the device using a blunt needle, removing excess fluid, and capping the remaining end with solid filament.

Prior investigations of Desai and colleagues focused on planar thin films of PCL fabricated by either spin- or slot-casting, which were then assembled into devices by heat sealing.^25−28^ While suitable for larger devices, a more refined fabrication process was required to miniaturize devices to a scale suitable for deployment by needle injection. Hence, a highly uniform and reproducible tubular construct with minimal wall thickness was required. Dip-casting was identified as a commercially established manufacturing method for medical products (e.g., catheters) that is capable of highly uniform and thin polymer coatings.^33^ To enable dip-casting, a custom apparatus was constructed to control of dip-casting draw speed and mandrel rotation. Figure 1 shows the process flow of dip-casting PCL thin films, loading tubular devices with either a compressed solid pellet or liquid, and sealing devices to maintain rounded or cylindrical ends.

**Figure 2:**
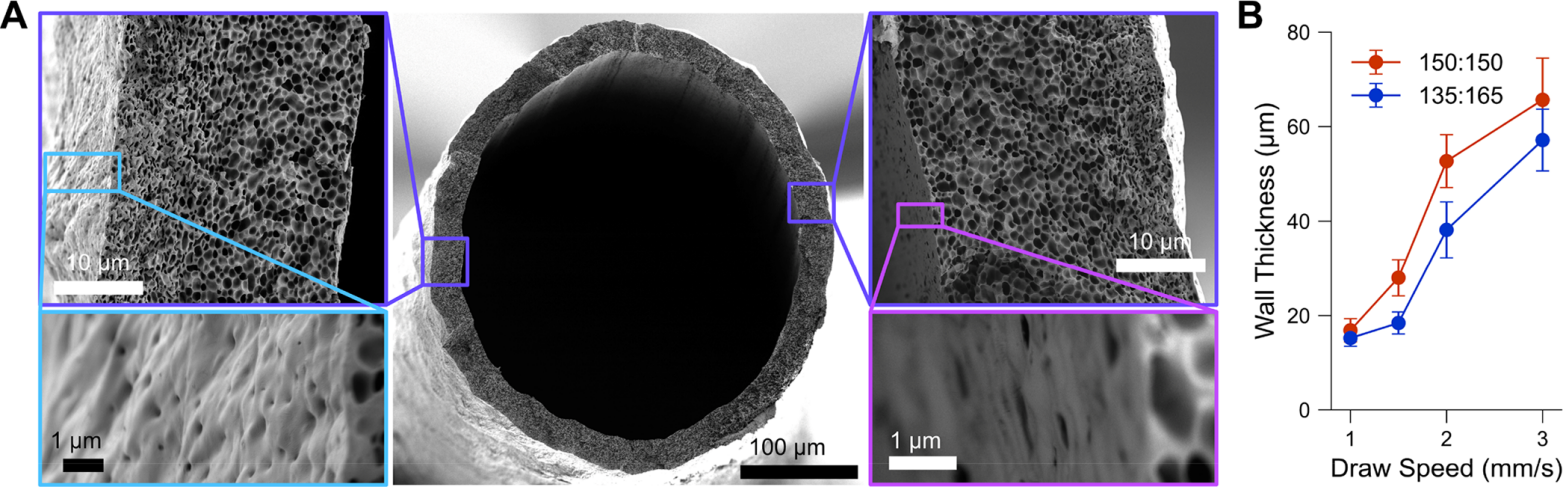
Dip-cast tube characterization. (A) Cross section SEM of a prototypical dip-cast tube, with insets showing finer membrane structure as well as exterior and interior surfaces. (B) Dip-cast tube wall thickness as a function of mandrel draw speed for select PCL:PEG solution compositions with qualitatively similar viscosities.

Solutions of PCL and poly(ethylene glycol) (PEG) dissolved in 2,2,2-trifluoroethanol (TFE) were used for dip-casting tubes, and stainless steel wires were used as mandrels for casting. On drying, phase separation of PCL and PEG occurs, and aqueous removal of the PEG phase results in a porous PCL tube. Porosity of materials cast from PCL:PEG solutions depend on the composition and molecular weights of the polymer constituents, and conditions of greatest interest are typically determined empirically. Figure 2 shows the correlation of tube wall thickness to the speed a mandrel is drawn from a PCL:PEG solution. In a simplified view, the dip-casting process is a competition between rate the mandrel draws viscous solution from the source and gravity countering that draw, where evaporative drying yields a solid film. At very slow draw speed, gravity dominates over the viscous drag of the mandrel drawing up material and yields a thinner coating. With increasing draw rate, polymer solution is drawn up by the mandrel and can evaporatively dry more quickly than gravity can drain the polymer solution away. When draw rate was increased in excess of 4 mm/s, resulting coatings were more prone to nonuniformity, exhibiting thickness variability in the form of periodic distortions that can resemble a “barber pole” pattern (Figure S1A). Without mandrel rotation a similar asymmetry was observed at all draw speeds and was attributed to fume hood airflow. For rotation speeds greater than 15 rpm, early investigations lacked a notable dependence of mandrel rotation on tube quality, so this parameter was not investigated exhaustively. A 30 rpm rotation speed was used in these studies to mitigate asymmetries and was found effective for the range of draw speeds and solutions tested.

When coating mandrels, there is an inherent trade-off between good coating adhesion and ease of coating removal. Commercially available poly(tetrafluoroethylene)-coated mandrels were investigated, but variability in coating adhesion during casting proved problematic. Further, it was not found to consistently improve coating removal to justify their use. Instead, stainless steel wires were used for coatings. Because bare stainless wires exhibited greater coating adhesion, it was necessary to further process the coatings prior to removal: a combination of bath sonication and the use of a surfactant solution (1% Tween 20) improved removal of coatings to yield non-deformed tubes. Even so, below a critical coating thickness (approximately 20 μm) coatings were prone to compress the tube onto itself when removed, yielding an accordion-like tube (Figure S1B). A variety of tube compositions and wall thicknesses were investigated and it was found that tubes drawn at 1.5–2 mm/s with PCL:PEG ratios from 1:1 to 1:1.3 were the most suitable for ease of handling and device fabrication. Given a focus on devices for 22G thin-walled needles or smaller, typical tubes were fabricated using a 14 mil (353 ± 4 μm) stainless steel mandrel with a typical outer diameter ranging from 30 to 40 μm based on membrane composition and casting conditions.

### Pelleting Solid Protein

A significant challenge in miniaturizing protein-loaded devices is achieving a high-density payload that can be readily incorporated into a device. Using a 14 mil tube described previously, the available payload volume of an idealized 10 mm long device is a scant 1 μL. Either a drug of interest must be highly potent or the formulation must optimize protein density within the available volume. For such reservoir devices, a liquid payload can be loaded by syringe with relative ease, yet achieving a high-quality seal with a cylindrical end proves more demanding. Alternatively, device sealing with a solid payload is simplified, but generating high density protein pellets at sub-mm scale is exacting. For storage and stability considerations, a lyophilized protein formulation is likely preferred and was a significant focus of this work. As-prepared lyophilized protein is largely empty space associated with the water removed from the formulation during drying, so lyophilized cake should be compressed to obtain the greatest payload within a miniature device. In addition to the fine scale these devices require, static can further exacerbate handling of these lyophilized products.

**Figure 3:**
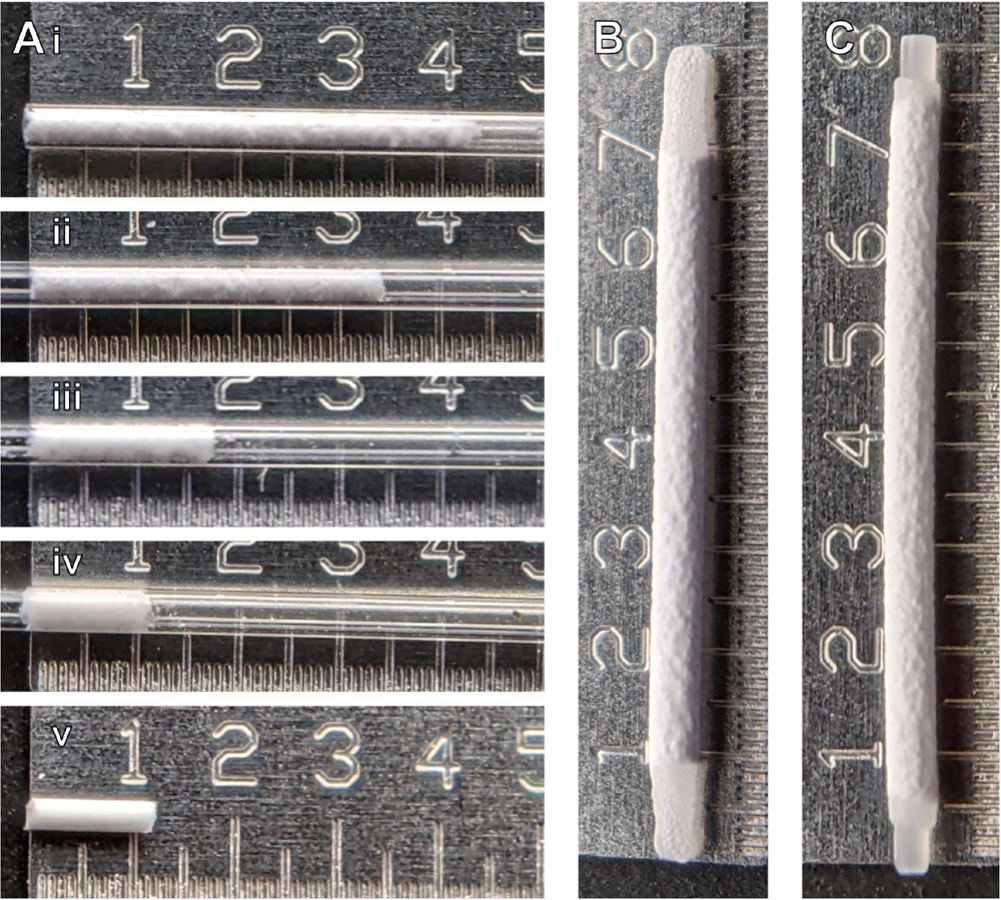
Prototypical components and devices. (A) Pelletization of lyophilized protein, showing (i) uncompressed protein as initially tamped into capillary, (ii) one-sided compression, (iii) two-sided compression, (iv) two-sided compression with rigid sleeve, and (v) the resulting pellet. Prototypical (B) self-fused cap and (C) solid-plug cap devices. Scale of ruler is mm in all images.

Lyophilized protein with minimal additives is generally hygroscopic and will consequently tend to naturally bind together when compressed. Given the challenges of machining at the scale of hundreds of microns, the pelleting approach here utilized commercially available capillaries with a well-defined inner diameter (300 μm) to form protein pellets. Protein could be loaded into the capillary by tamping the capillary into raw lyophilized cake. At this point, the protein is still largely uncompressed, so nitinol wires were introduced to both ends of the capillary and hand pressed to generate a consistent pellet. Across a series of devices, pellet density for pure bovine-serum albumin (BSA) was found to be 1.05 ± 0.15 g/cm^3^ using an optimized compression method (calculated from 6 sets of device with 26 total devices). Lyophilized protein alone was often found to bind in the capillaries preventing removal. To allow easy pellet removal, capillaries were precoated with magnesium stearate powder, a common solid-state lubricant.^34^Figure 3 shows an example of lyophilized protein within a capillary as initially loaded, through the phases of compression, and following removal from the capillary. To assess the impact of pelletization on dissolution of the protein, pellets were incubated in phosphate-buffered saline (PBS) and dissolution was tracked. Within 20 min, pellets were consistently fully dissolved, indicating that dissolution limited release is not expected given excess of media.

### Loading and Sealing PCL Tubes

In prior work, sealing PCL devices employed a heat source and pressure applied perpendicularly to two films to melt and fuse the material. To seal the ends of tubular PCL devices, this paradigm is impossible without flattening and widening the tube at the seal location, which would prevent easy insertion and deployment via needle (Figure S1C). Fortunately, the small diameter of these tubes allows direct heating at the tube end to allow the tube to naturally collapse and melt to itself. While simple in premise, this approach is extremely challenging to accomplish in a controlled and reproducible way at the lab scale. Spatial orientation, heating intensity, and consistency of the heating source all play a role in self-seal quality, and orientations that allow easy microscopic visualization can result in bending or sagging due to gravity. A self-sealing method does minimize the polymer required to seal, but without precise heat exposure one can expect inconsistency in the dimensions and quality of the fused end.

Alternatively, device ends can be capped using a solid plug of PCL. A PCL plug with similar size to the tubular opening was found difficult to insert and achieve a consistent high-quality seal. An approach was developed to allow easy insertion of a solid PCL segment that would expand to fill the tube end upon heating. This method took advantage of PCL necking upon plastic deformation: PCL filaments roughly the inner diameter of the tube could be plastically deformed to decrease their diameter and correspondingly increase their length. For instance, a PCL filament with diameter 350 μm can be necked by hand to a diameter of 150 μm. Such a necked filament can be cut to approximately 1–3 mm and easily placed within the open device end. Upon heating, the necked filament relaxes toward its pre-deformed state with an accompanying decrease in length and increase in diameter.^35^ With this method, the heat source can be positioned further from the tubular material and the filament acts as a thermal sink for the heating element. The melted filament gently fuses with the tube and precise fitting of the filament within the tube is not required. This method is suitable for both liquid- and solid-loaded devices and is strongly preferred for a high-quality seal with liquid-loaded devices. Since a filament displaces fluid within the lumen of a device, it also provides a tool to remove excess fluid prior to sealing. Excess filament can be pushed within the tube and the displaced excess fluid can be removed. Next, the filament can be withdrawn from the tube to its final position, where the remaining liquid payload retreats by capillary action to the space previously occupied by the filament. Following this sequence, the location of the filament-tube seal is effectively dry, and reliable polymer–polymer contact is possible without impact from the liquid payload. With either liquid or solid payloads, excess PCL from the plug can be further cut down or adapted for device manipulation as needed. Such filament seals can yield very uniform seals even without precise heating, but the extent of the filament and exact location within the tube can be hard to gauge given these porous tubes are opaque.

### Deployment of Devices

**Figure 4:**
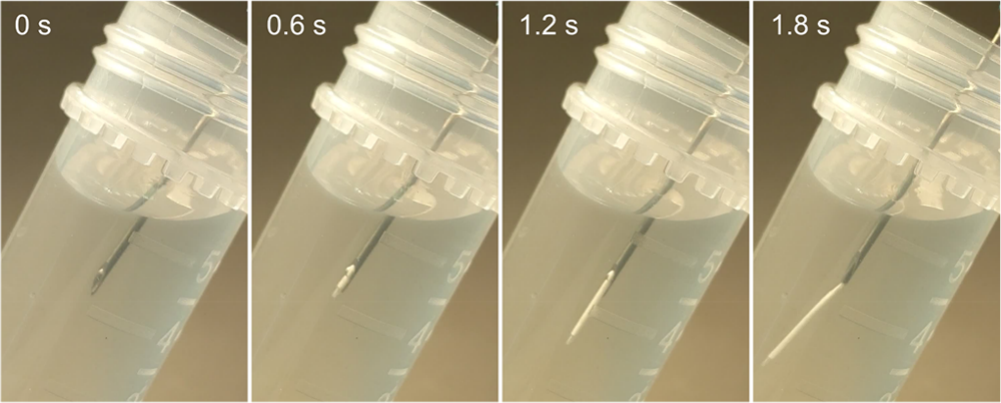
Injection sequence of a placebo device into aqueous media.

Such devices can be deployed by 22G thin-walled needle. In general, either fluid (e.g., PBS) or a rigid pin can be used to expel device from needle (as in Figure 4; Movie 1). The placebo device utilized here is empty (i.e., air-filled), so it is buoyant in media. When deploying with fluid, additional tolerance between the needle and device is generally required to avoid friction between the device and needle bore.

### In Vitro Release

**Figure 5:**
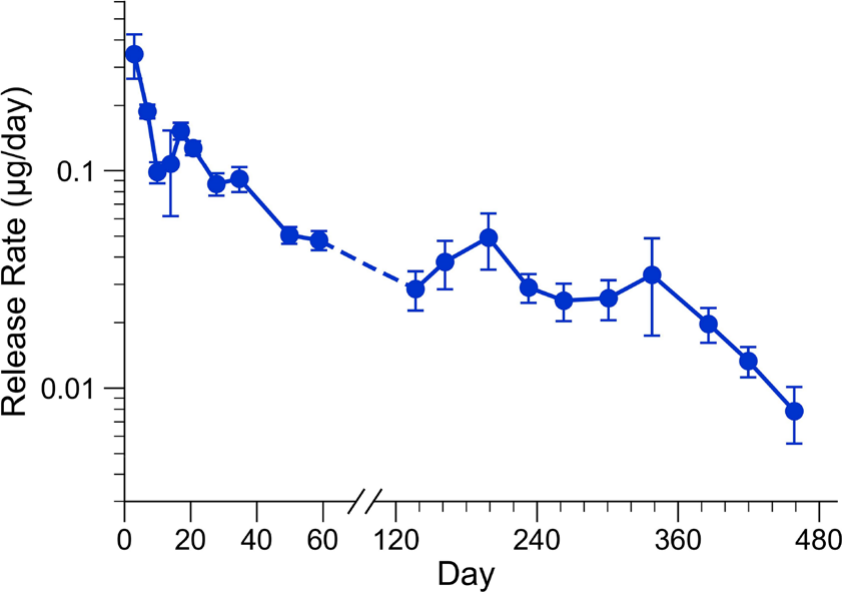
Long-term in vitro release from a solid-loaded PCL device. Devices had a porous area of 7.9 ± 0.9 mm (overall dimension 10.7 ± 0.9 mm) and were loaded with 367 ± 133 μg of BSA (400 ± 145 μg total payload; *n* = 4). Devices were sampled periodically where all media was exchanged.

Figure 5 shows release of BSA from prototypical devices over the course of 60 weeks. The BSA used for the pelleted payload was prepared from a solution of BSA in PBS with PEG3350. PEG was included to act as a protein solubility adjunct as outlined in Schlesinger et al.^26^ Devices were loaded with an overall payload of 400 ± 145 μg, which corresponded to a BSA loading of 367 ± 133 μg for this formulation. Device porous length was 7.9 ± 0.9 mm with a corresponding external porous surface area of approximately 1 mm^2^. One device was excluded from the data set as it had an abrupt increase in release at day 94, which was attributed to damage during a media exchange (complete data set in Figure S2). A subset of the samples collected could not be quantified as the sampling interval was insufficient to accumulate sufficient protein for quantification by micro-BCA protein assay kit; consequently, a conventional plot of cumulative release cannot be calculated.

### Multidevice Injection Safety

**Figure 6:**
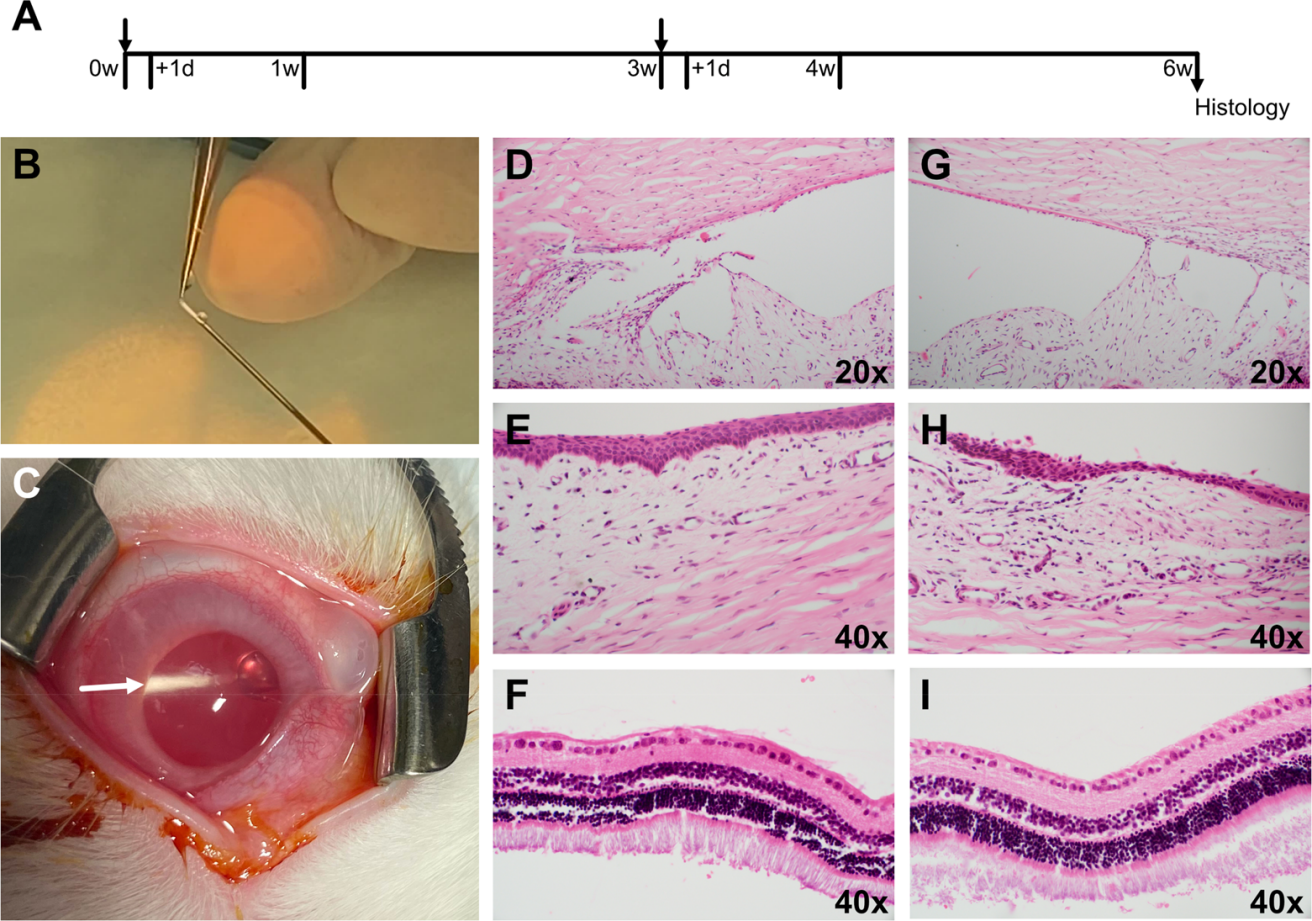
Repeated injection study. (A) Timeline for injection of multiple devices, with injections at *t* = 0 and 3 weeks. (B) Manual insertion of device into bore of needle. (C) Device after placement by injection (white arrow pointing to device in vitreous). Representative histopathology images for (D–F) control eyes receiving a sham injection and (G–I) eyes receiving two injected devices, where tissues were stained with hemolysin and eosin as follows: (D,G) endothelium and angle, (E) Perilimabl region, (H) limbux with perilimbal inflammation, (F,I) retina.

Drug delivery devices fabricated from PCL have the advantageous property of maintaining their structural integrity for a majority of their degradation;^24^ however, when applications require repeated administration, a new device must be placed while an empty device shell is undergoing the final stages of degradation. Given the potential of such miniaturized devices in ocular applications and a limited ocular volume, it is valuable to gauge the impact of repeated injection with multiple devices present. Figure 6 shows a repeated device injection sequence to assess tolerability of multiple devices within the vitreous cavity using a rabbit model. The sequence included intravitreal injection of a first device followed by injection of a second device 3 weeks later to simulate the process of delivering a fresh device while a device is still present within the vitreous. To avoid any artifacts of including an API, placebo devices were used, where a solid PCL rod was used as the placebo payload. Examinations included evaluation of the conjunctiva, cornea, anterior chamber, lens, vitreous, and retina as well as measurement of the intraocular pressure. Postinjection examinations revealed that the devices were well tolerated by the rabbit eyes. There was no significant inflammation noted on any of the follow up exams. Local irritation (conjunctival injection and chemosis) was noted after three out of the four injections and all resolved by the week 1 exam. Human error and misdirection of the needle in one of the injections caused an injury to the lens capsule and the crystalline lens, which resulted in a focal cataract. Two devices can be seen in the vitreous at the end of the testing period. Tissue was extracted for histology at the end of 6 weeks (3 weeks following the second injection), and representative images are shown in Figure 6. Histopathology was notable for peri-limbal inflammation that is consistent with local mechanical trauma from device injection (Figure 6H). Notably, the corneal endothelium, retina, and choroid did not show any abnormalities.

## Discussion

Platform technologies for delivery of biologic therapeutics face many challenges, and molecule specific concerns make universal delivery solutions a challenging task. For any specific biologic, the primary, secondary, and tertiary structure may be susceptible to routes of degradation, like oxidation or deamidation, or a loss of activity by aggregation, all of which can be pH or concentration dependent behavior. A delivery modality that suitably disconnects the mechanism for controlled release from the composition and formulation of the protein can benefit from parallel optimization of device fabrication and therapeutic formulation. This methodology was employed throughout this work, yet prior work demonstrated that there is an inevitable coupling of device and formulation optimization.^26^ The device fabrication demonstrated could be generically applied, and release was simply demonstrated with the model protein albumin. Given an interest in ocular delivery, miniaturization and refinement of manufacturing techniques were a focus of this work, particularly looking toward the many translational hurdles. It would be straightforward to increase the dimensions of such devices to improve payload and extend release duration, but that was not the intent of this work.

For ocular delivery, Ozurdex, Iluvien, and the Port Delivery System provide a guide for acceptable intravitreal sustained delivery implants. Out of the three, only the PDS delivers a biologic payload. Given its recent voluntary recall, there is clearly a need for minimally invasive sustained drug delivery devices.^18,36^ Ozurdex and its corresponding 22-gauge thin-walled injector provide the current standard for maximum tolerated device size. Ozurdex and Iluvien have lengths of 6 and 3.5 mm, respectively, and the Port Delivery System has an overall length of 8.4 mm.^12,37,38^ Given these reference points, acceptable device length should be 8 mm or smaller, which is expected to avoid interference with the visual axis.

A number of device iterations employing thin-film PCL membranes for controlled release devices have been demonstrated over the past decade, yet a number of hurdles have limited their capacity for further development and translation. Planar geometries were suitable for surgical deployment but could not be fit into an acceptable needle size, even if furled into a cylindrical form as proposed in early work. Later embodiments that moved toward a cylindrical form relied on a complex assembly process, and the resulting device had a seam of excess material along the device length as well as ends that taper to a flattened and broader seal. The work of Waterkotte et al. improved on this to eliminate the seam at the cost of superglue inclusion. The work of Jiang et al. nicely demonstrated the use of electrospinning to yield high quality cylindrical constructs from PCL, and finally demonstrated the capacity for device injectability at the desired size scale. This was an important demonstration of the physical integrity of a tubular PCL device and a methodology to load such devices.

In the work of Jiang et al, the materials focused on compatibility with a 22-gauge thin-walled needle (OD < 500 μm) and produced tubes with a 260 μm inner diameter with a nominal wall thickness of approximately 90 μm. For Jiang et al., geometrically the wall accounts for 65% of the cross sectional area of a device fabricated from such tubes. In the work presented here, tube diameters as low as 30 μm were reliably fabricated and handled, so it was possible to cast tubes with a larger inner diameters (∼350 μm) while maintaining a 22-gauge needle criteria. Hence, geometrically the wall accounts for only 27% of the cross sectional area of a device fabricated from these tubes, which is a significant improvement over the prior work. Commercially available wire was used as the casting mandrel, which is available in thousands-of-an-inch increments. If the casting mandrel were machined more precisely, additional gains in tube fabrication could be possible with wall thickness and tube inner diameter optimized to the desired needle parameters. For in vivo implantation, devices were loaded by hand into 22-gauge needles, and one can expect a commercial product would utilize a preloaded injector, as with Ozurdex and Iluvien.

Given the goal of devices suitable for 22-gauge injection, such small devices must optimize their formulation density for long-term release. In this work, a method to pelletize protein was developed to yield pellets that could be suitably placed within PCL tubes. Since pellets need to slide into tubes, some tolerance was required between the tube inner diameter and pellet outer diameter to facilitate assembly, and improvements can be expected with advanced manufacturing fixturing and automation. In particular, robust fixtures capable of creating reproducible pellets will be essential to further development, along with refined handling methods. While directly loading lyophilized protein cake into PCL tubes was investigated, difficulty handling the lyophilized product, a lack of compression for the loaded material, and a propensity to damage the PCL tube on loading made this method unfeasible. Based on estimates of pellet density for pure protein, it is possible to achieve 76.5% of an upper limit for expected density (∼1.37 g/cm^3^).^39,40^ Assuming the cross section of the pellets developed here and the measured density, one can predict an approximate loading of 74 μg/mm pellet loaded. Assuming 7 mm of pellet can be loaded in an 8 mm long device, such a device would have an overall loading of just 520 μg. When considering anti-VEGF therapies, bolus dosing requires 0.5, 1.25, and 2 mg for ranibizumab, bevacizumab, and aflibercept, respectively. Sustained delivery is expected to improve the utilization of an equivalent payload, but devices miniaturized for 22-gauge or smaller injection may simply lack the required payload for meaningful therapy. For instance, in Schlesinger et al. a target release for aflibercept devices was specified as 30 μg/day: a 520 μg payload would last just 17 days. Thus, it is essential to thoughtfully interrogate the actual required release rates and the basis for their selection. In particular, it will be essential to reconsider how the release profile of a sustained delivery device can be advantageous over that of a conventional bolus dose at the core of most dosing estimates. Adoption of these devices may prove more attractive if the sustained-release behavior of such therapeutics is better understood since most targets of release rate are based on evidence from trials employing bolus delivery. Even the Port Delivery System platform release is fundamentally first order in nature, so gains may yet be attainable for a device exhibiting zero- or pseudo-zero-order release. Further, combining such a device with an initial bolus loading dose would be a plausible methodology to extend the duration of therapy. It is apparent that miniaturized devices of this type will presumably require prospective APIs with at least modest potency to be attractive for development.

Experiments to gauge dissolution of pelleted protein observed complete dissolution with relative ease; however, excess media was used in these experiments and the solution was agitated on a shaker plate. Hydration of the protein pellet within a device is expected to be considerably slower given aqueous permeability of the membrane and a high reservoir protein concentration. Given protein specific behavior at high concentrations, further investigation will be required with for a specific candidate API. PEG-mediated protein solubility reduction as demonstrated in Schlesinger et al. or alternate formulation methodologies may be required to retain acceptable protein stability over long durations.^26,41^

Lastly, achieving sealed devices that maintain a cylindrical cross section without flattening and widening the device at the ends is of utmost importance for injectability. With suitably thin polymer membranes, self-collapsing seals may be suitable if processing can be effectively standardized. In practice, lab-scale fabrication with this method was found to be error prone for maintaining cylindrical devices, if albeit otherwise producing a suitable seal. Alternatively, sealing with a solid plug of polymer was found to be effective and suitable for any type of loading, and the excess material introduced is expected to be tolerable, especially once further optimized.

Applications in the eye place very specific constraints on device size to avoid obscuring the visual axis while remaining injectable by a needle gauge known to result in a self-sealing wound. Miniaturized devices placed by injection have evident applications elsewhere in the body, yet each prospective location will present unique design challenges and requirements (e.g., mechanical force within joints or highly vascularized nasal tissues). Elsewhere in the body, device size and overall therapeutic payload for such devices may benefit from an increased spatial dimension, but the strength of this approach remains localized delivery since systemic delivery will only be possible with the most highly potent of therapeutics.

## Conclusion

This work demonstrated the fabrication of devices utilizing dip-casting to produce miniaturized tubular materials. By employing a commercially established and trusted process employed in common fine-scale medical products (e.g., vascular balloons), the translational route for manufacturing development follows a more tried route. Through process optimization, wall thickness was reliably produced for long segments of tube, which can help maximize the payload contained in a reservoir-based device. For applications in injected devices, maintaining cylindrical devices suitable for deployment by injection has been a challenge not clearly overcome by prior attempts with heat-sealed polymer forms. This work demonstrates two methods to seal device reservoirs while maintaining a fundamentally cylindrical form factor. With ocular applications prominent for such miniaturized devices, devices produced here were successfully deployed by 22-gauge thin-walled needle, and devices were well-tolerated in a rabbit model for device injection. Further, repeated injection and simultaneous residence of multiple devices within the rabbit eye were shown to be safe. Given the potentially long lifespan for PCL-based materials, devices fabricated in this method were able to achieve sustained release of protein for >400 days. Based on loading estimates for the fabrication methods herein, the payload of a clinically acceptable device is low compared to bolus dosing. If such devices are to make inroads, a reexamination of the basis for dosing and the important distinction between a bolus dose and long-term sustained release will be essential in determining their applicability.

## Experimental Section

### Dip Casting Tubes

Polycaprolactone (80 kDa), polyethylene glycol (2050 Da), and 2,2,2-trifluoroethanol were all obtained from Sigma-Aldrich (St. Louis, MO). Magnesium stearate was obtained from Spectrum Chemicals (New Brunswick, NJ).

To prepare PCL-porogen solutions, PCL and PEG were weighed and TFE was added to achieve the desired concentrations of polymer in solvent (denoted at mg polymer per ml of neat solvent). Solutions were mixed using a Tube Revolver (part 88881001; Thermo Scientific, Rockford, IL) until fully dissolved.

A custom dip-casting apparatus was built for controlled casting of PCL tubes. A 300 mm Linear Stage Actuator (part 101-80-125; Sainsmart, Lenexa, KS) was used to control the rate mandrels were drawn out of the PCL solutions, and an additional NEMA-17 stepper motor (part 324; Adafruit Industries, New York, NY) was attached to the stage platform to control the rotation of the mandrel. Each motor was driven by an Arduino Uno microcontroller (Arduino,, Turin, Italy) and a dedicated TB6600 Stepper Motor Driver (part 101-60-197; Sainsmart, Lenexa, KS; part B07PQ5KNKR; Twotrees, ShenZhen, China). An Arduino-compatible LCD keypad module (part 101-50-112; Sainsmart, Lenexa, KS) was used for user input. Source code may be found at https://github.com/dabernards/dip-cast-controller.

Matte-finish stainless steel wires (OD = 0.014″, part 6517K63; McMaster-Carr, Elmhurst, IL) were used as mandrels for dip-casting, and small hole drill chuck adapters (part 30505A5; McMaster-Carr, Elmhurst, IL) were used to hold mandrels and interface with the coupling to the rotating stepper motor. Wires were cut to length and annealed at 350 °C for 30 min before casting, which was found to improve removal of coatings relative to as-received wires. All coatings were cast at 30 rpm with a draw rate between 1 and 3 mm/s. To remove coatings, mandrels were sonicated in a VWR model 75T Sonicator (Radnor, PA) for 10 min and subsequently mechanically removed. Resulting tubes were then rinsed with deionized water and allowed to soak further in fresh deionized water overnight on a tube rotator. Tubes lumens were then blown dry using a blunt needle and syringe, and the tubes were further dried under house vacuum.

### Fabrication of Devices

To fabricate liquid-loaded devices, tubes were first capped with a solid PCL filament. PCL filament was produced using a custom ram-based hand extruder. The extruder utilized commercial orifices (part 2822T18-0.01”; McMaster-Carr, Elmhurst, IL) and stainless steel tubing and fittings (parts 4464K351 and 9157K49; McMaster-Carr, Elmhurst, IL). A shaft collar (part 6436K52; McMaster-Carr, Elmhurst, IL) was used to facilitate fixturing in a conventional clamp. Temperature control was achieved using heating tape (part FGS0031-010; Omega Engineering, Tarzana, CA) attached to a temperature controller (model CN1A; Omega Engineering) with a J-type thermocouple, where the thermocouple was placed between the heater tape and stainless tubing. A temperature of 110 °C was empirically determined to achieve 350 μm diameter filaments suitable for capping tubes cast on 14 mil mandrels. To cap tubes with PCL filament, first filament was stretched and cut to length. The deformed filament was then inserted into the tube and heated using a high temperature cautery kit (model DEL1; Bovie Medical, Clearwater, FL), where the melted filament adhered to the tube with minimal heating. With one end sealed, tubes were cut to length, and protein solution was loaded with a blunt needle and syringe. Next a second filament piece was inserted into the loaded tube and excess protein solution was removed. While excess solution was removed, the filament was placed further into the tube than its final position; the filament was then retracted to its final position and sealed with the hand-held cautery tool as before. Upon retracting the filament, the protein solutions retreats within the tube and ensured good polymer–polymer contact upon sealing.

Formulation was prepared by dissolving BSA in deionized water. Once fully dissolved, PEG3350 was weighed and added and 10X PBS was added by volume to achieve a composition of BSA, PBS, and PEG3350 of 91.7%, 1.4%, and 6.7%, respectively. The resulting formulation was then dried using a VirTis Advantage Plus Freeze Drier/Lyophilizer (SP Scientific, Stone Ridge, NY). First, samples were flash frozen by submerging in liquid nitrogen and then attached to an external jar for drying in the lyophilizer’s manual mode.

To fabricate solid-loaded devices, tubes were similarly capped as with liquid-loaded devices. In addition to the filament sealing approach, solid-loaded device were able to be self-sealed by heating the tube alone and having it fuse itself as it collapses. Next, pellets were prepared by loading in glass capillaries with 300 μm inner diameter (CTech Glass, River Edge, NJ). Capillaries were first coated with magnesium stearate to act as a solid-state lubricant. The capillary was then tamped into a vial of lyophilized protein, and the protein was compressed within the capillary using 11 mil nitinol wire (Malin Co., Brookpark, OH). Given the fine gauge of the capillaries used, a rigid support around the capillary was used to improve the consistency of the compression. Generally pellets of 1–2 mm in the length were used in device fabrication. Unlike liquid-loaded devices that were roughly cut to the desired size prior to loading, longer tubes were used for solid loading to enable easier loading of protein pellet. Prior to loading, fine tweezers were placed within the lumen of the tube to gently increase the tube bore at the opening. Pellets were then loaded and tubes were cut to leave some overhang beyond the pellet position. Devices were then sealed with either the self-fusing or filament capping method.

To fabricate devices for serial injection of multiple devices in vivo, a core of PCL filament was used as the device core. Devices were sealed as with liquid-loaded devices.

### Imaging Miniaturized Devices

Photos of pelleted protein and devices were taken with a Moment 10X Macro lens for use with a conventional smart phone. For video, ffmpeg was used to crop video and extract still images. A Micro-Ruler with mounted handle (part 13635; Ted Pella, Redding, CA) was used to provide scale in photos, with millimeters numbered and subdivisions denoting 0.1 mm.

### In Vitro Elution Studies

The primary method to gauge device performance was through elutions studies. Elution studies were performed by placing devices in a vial in 0.5 mL PBS and incubating at 37 °C on a shaker plate at 100 rpm. To avoid artifacts from residual protein or failed devices, media was added to each device, which was then vortexed for 5 s to rapidly remove any surface adhered protein present from the fabrication process while avoiding capturing the initial device release behavior. In addition, the aggitation of the vortexing process provided a simple assessment of device integrity since any weak seal or joint would be likely highlighted in this process. Then elution samples were collected by removing all media, replacing with fresh media, and storing the collected sample at 4 °C until assay. Protein concentration was quantified by micro-BCA protein assay kit following the kit instructions (Thermo Scientific, Rockford, IL).

To assess the dissolution of compressed protein pellets, a dissolution study was performed. Pelleted protein was generated as described above. Pellets of known mass were added to vials with PBS and were subsequently incubated. Performing a complete media exchange was found to be too error prone, so 25% of the media was removed and fresh media added to replace it. Samples were collected at 5 min intervals to measure the dissolved protein content over time. The dilution associated with media removal/replacement was accounted for when calculating the total mass dissolved.

### Scanning Electron Microscopy

To quantify tube wall thickness and morphology, samples were prepared for scanning electron microscopy. Tubes were dipped in liquid nitrogen and were freeze fractured. Samples were mounted with 90° sample holders using Pelco colloidal graphite with isopropanol base (part 16053–20, Ted Pella, Redding, CA). All tubes were coated with 3 nm of Au/Pd by a Leica EM ACE600 sputter coater and imaged with a Carl Zeiss Sigma 500 field emission scanning electron microscope using a secondary electron detector.

### Serial Injection of Devices In Vivo

Devices were injected into rabbit eyes in accordance with the ARVO Statement for the Use of Animals in Ophthalmic and Vision Research and under UCSF IACUC protocol AN184325–01. After adequate anesthesia, placebo devices were loaded into a 22-gauge thin-walled needle (part 16-N221; McKesson, Irving, TX) prefilled with sterile water and then injected into the anterior chamber through the superior limbus. A site 2.5 mm posterior the limbus at 12 o’clock was marked and device was injected at the marked site in a direct approach without a scleral tunnel. If the device refluxed back or was unsuccessful, the above steps were repeated immediately at a location 2 clock hours away from the initial site. Sham injections without the device were performed, where 0.1 cc of balanced salt solution (BSS) was injected. Devices were injected at 3 week intervals, and two devices were injected into each rabbit’s right eye with the left eye serving as control.

Animals were given mild sedation for routine exams. Baseline anterior segment examination was performed with 20 diopter indirect biomicroscopy lens (Volk Optical Inc., Mentor, OH) with topical anesthesia (proparacaine 0.5%) and dilation (2.5% phenylephrine hydrochloride and 1% tropicamide). IOP measurement was performed prior to device implantation using a tonopen. Posterior segment exam was also performed with a 20 diopter lens using an indirect ophthalmoscope. Eyes were examined at 1 day, 1 week, and 3 weeks following each injection. For postinjection day 1 examinations, the eyes were not dilated.

At 6 weeks following the initial injection (3 weeks after the second injection), animals were euthanized. Anterior and posterior segment examinations (with dilation) were performed prior to euthanasia. Globes were enucleated immediately following euthanasia using Westcott scissors and forceps. Whole globes were fixed in 10% neutral buffered formalin and refrigerated until histology was performed.

Formalin-fixed enucleated eyes were bisected then processed for paraffin embedding. Ten micron sections were cut vertically through the pupillary optic nerve and stained with hematoxylin and eosin. The corneal epithelium, corneal stroma, endothelium, trabecular meshwork, vitreous, uvea, and retina were imaged.

## Supporting Information Available

Alternate tube morphologies; in vitro release showing individual device elution (PDF)
Movie of device injectability into PBS (MP4)

## Abbreviations

API: active pharmaceutical ingredients
PDS: Port Delivery System
PLGA: poly(lactic-*co*-glycolic)
PCL: poly(caprolactone)
PEG: poly(ethylene glycol)
TFE: 2,2,2-trifluoroethanol
BSA: bovine-serum albumin
PBS: phosphate-buffered saline
BSS: balanced salt solution
