# Supplementary material for: Injectable Devices for Delivery of Liquid or Solid Protein Formulations
Source: ACS Mater Au. 2023 Mar 13;3(3):255–64. doi: 10.1021/acsmaterialsau.3c00004 (PMC10176615; doi:10.1021/acsmaterialsau.3c00004)
Supplement: Supplementary file 1 — mg3c00004_si_001.pdf [file mg3c00004_si_001.pdf]

Supporting Information for:

Injectable devices for delivery of liquid or solid protein formulations

Daniel A. Bernards[1], Chu Jian Ma[2], Youning Zhang[2], Tannia M. Rodriguez[1], John Dickson[2], Bhushan N. Kharbikar[1], Robert B. Bhisitkul[2], Tejal A. Desai[1,3*]

[1]University of California, San Francisco, Department of Bioengineering and Therapeutic Sciences, San Francisco, CA 94143, USA

[2]University of California, San Francisco, Department of Ophthalmology, San Francisco, CA 94143, USA

[3]Brown University, School of Engineering, Providence, RI 02912, USA

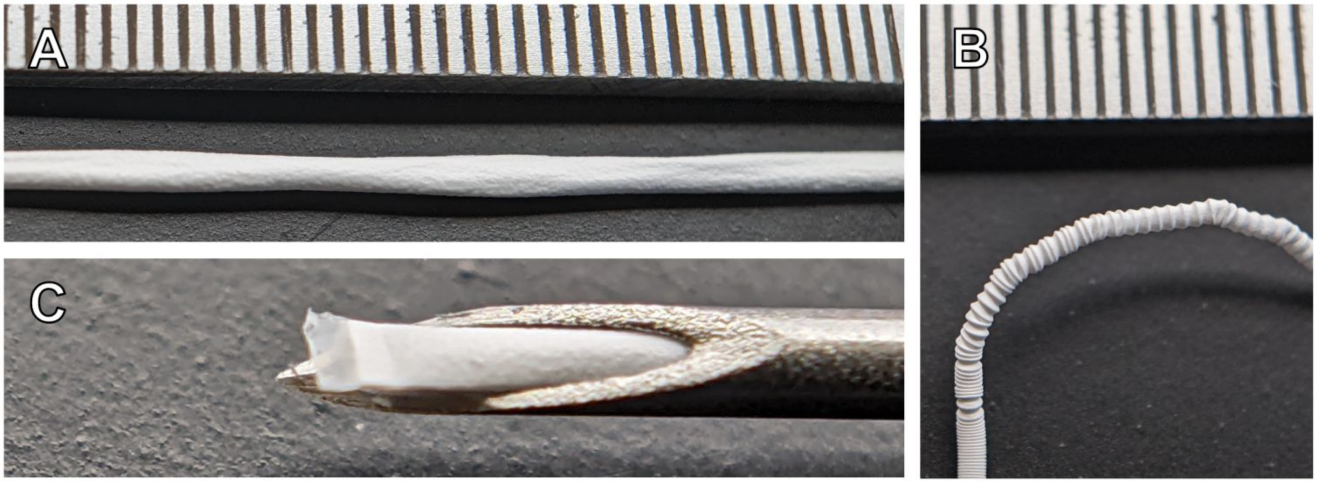

Supplemental Figure 1: Alternate tube morphologies. (A) Barber-pole type tube defect resulting at faster draw speeds. (B) Compressed accordian-type tube defect resulting from poor release from casting mandrel. (C) A tube capable of insertion into a 22-gauge needle with a single flat crimp-type seal and associated limitations inserting fully into needle bore.

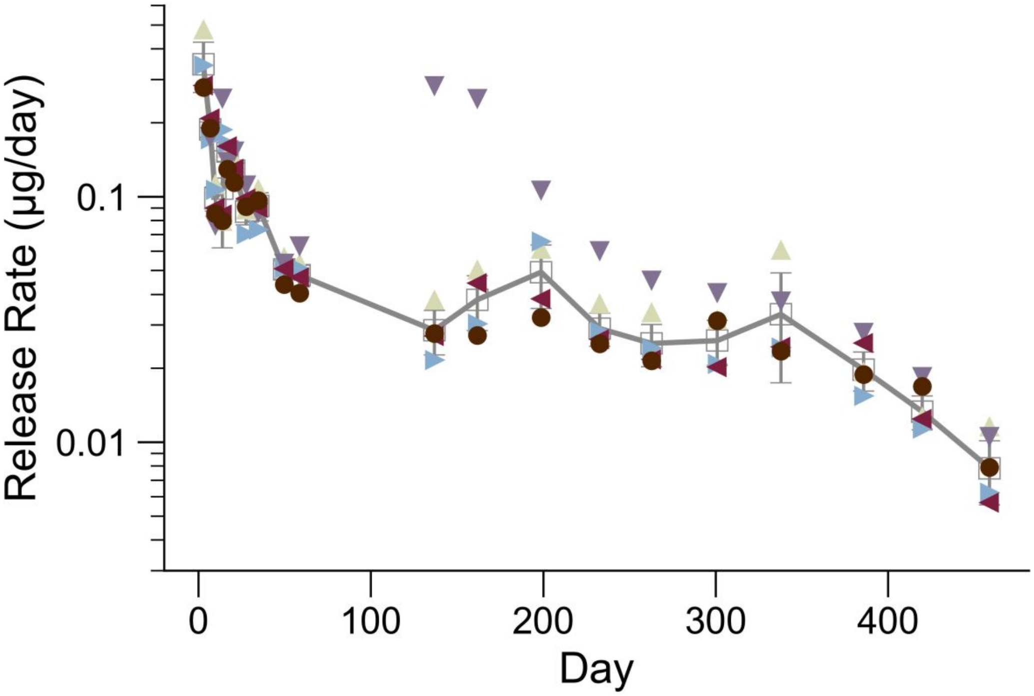

Supplemental Figure 2: Long-term in vitro release from solid-loaded PCL devices, where individual device release is shown. Excluded outlier is purple inverted triangles, and average release (gray line) excludes outlier.
